# Supplementary material for: The Importance of Allelopathic Picocyanobacterium Synechococcus sp. on the Abundance, Biomass Formation, and Structure of Phytoplankton Assemblages in Three Freshwater Lakes
Source: Toxins (Basel). 2020 Apr 16;12(4):259. doi: 10.3390/toxins12040259 (PMC7232185; doi:10.3390/toxins12040259)
Supplement: Supplementary file 1 [file toxins-12-00259-s001.pdf]

Supplementary Materials: The Importance of Allelopathic Picocyanobacterium *Synechococcus* sp. on the Abundance, Biomass Formation, and Structure of Phytoplankton Assemblages in Three Freshwater Lakes

Iwona Bubak, Sylwia Śliwińska-Wilczewska, Paulina Głowacka Agnieszka Szczerba and Katarzyna Możdżeń

Table S1. List of species detected in three lakes (ŁL, RL, and ŻL) in study period.

Present Taxa	ŁL				RL				ŻL			
	V	VI	VII	VIII	V	VI	VII	VIII	V	VI	VII	VIII
**Cyanophyceae**												
Anabaena sp.	■	■	■	■		■	■	■				
Aphanizomenon flosaquae	■		■	■	■	■	■	■	■	■	■	
Aphanizomenon sp.												■
Aphanocapsa delicatissima	■		■	■	■			■	■	■	■	
Aphanocapsa holsatica	■	■	■	■	■	■	■	■	■	■	■	
Aphanocapsa incerta	■		■	■	■	■	■	■	■	■		■
Aphanothece clathrata		■	■			■	■			■		
Aphanothece parallelliformis		■			■	■		■	■			
Cyanodictyon imperfectum				■					■			
Cyanodictyon planctonicum	■		■									
Cyanonephron styloides								■				
Lemmermanniella pallida			■	■			■		■			
Limnothrix redekei			■		■			■	■	■	■	
Merismopedia punctata				■		■						
Merismopedia tenuissima			■									
Microcystis sp.						■	■					
Planktolyngbya limnetica	■	■		■			■			■	■	
Snowella atomus			■	■		■		■	■			
Snowella lacustris		■				■	■			■		
Snowella septentrionalis	■		■		■	■	■	■	■			
Woronichinia compacta											■	
Woronichinia naegeliana	■									■		■
**Cryptophyceae**												
Cryptomonas erosa	■	■	■	■		■			■	■	■	■
Cryptomonas ovata					■	■			■			
Rhodomonas lens										■		
Rhodomonas sp.	■	■	■	■	■	■				■		■
**Dinophyceae**												
Amphidinium crassum	■									■		
Ceratium hirundinella	■	■	■	■	■	■	■	■			■	
Gymnodinium sp.				■		■				■	■	
Gyrodinium helveticum									■			
Gyrodinium sp.			■						■			

Peridiniopsis cunningtonii										■		■
Peridinium aciculiferum						■			■			
Peridinium gatunense												■
Peridinium willei					■							
Protoperidinium bipes						■			■	■	■	
**Chrysophyceae**												
Dinobryon divergens	■		■	■	■	■		■	■	■	■	■
**Synurophyceae**												
Mallomonas caudata	■					■		■	■	■	■	■
**Bacillariophyceae**												
Amphora pediculus				■								
Amphora sp.						■						
Aulacoseira granulata		■										
Aulacoseira italica	■							■			■	
Cyclotella comensis					■							■
Cyclotella cyclopuncta					■							■
Cyclotella meneghiniana									■			
Cyclotella pseudostelligera				■								
Cyclotella radiosa	■	■	■	■	■	■	■		■	■	■	
Cyclotella stelligera				■								■
Cymbella cistula	■	■	■									
Cymbella helvetica											■	
Cymbella silesiaca		■										
Epithemia adnata			■	■								
Epithemia sorex		■										
Fragilaria capucina v. *acuta*								■				
Fragilaria crotonensis				■					■	■	■	
Gomphonema truncatum		■										
Gyrosigma acuminatum								■				
Melosira moniliformis		■										
Navicula heimansioides	■							■				
Navicula radiosa								■				
Navicula sp.										■		
Navicula sphaerophila	■											
Nitzschia acicularis	■		■									
Nitzschia paleacea	■									■		
Ulnaria delicatissima	■						■					
**Charophyceae**												
Cosmarium bioculatum	■						■	■			■	■
Cosmarium punctatulum							■					
Elakatothrix genevensis		■	■									
Koliella longiseta	■			■	■		■	■	■		■	■
**Euglenophyceae**												
Anisonema acinus												■
Euglena sanguinea												■
Eutreptiella spp.				■							■	
Monomorphina pyrum												■
Patelomonas sphagnophila											■	■
Trachelomonas hispida									■			■

Trachelomonas sp.												■
Trachelomonas volvocina											■	
**Chlorophyceae**												
Actinastrum hantzschii		■	■	■	■			■		■		
Ankistrodesmus falcatus	■											
Ankyra lanceolata		■						■				
Chlamydomonas sp.				■								
Chloromonas sp.			■		■		■			■	■	
Coelastrum astroideum												■
Coelastrum microporum				■				■				■
Crucigenia tetrapedia		■		■		■					■	■
Crucigeniella apiculata							■	■				
Desmodesmus communis	■						■	■	■	■	■	■
Desmodesmus intermedius											■	
Dictyosphaerium pulchellum	■						■					
Eutetramorus fottii		■	■	■	■			■			■	■
Lagerheimia genevensis									■			
Monoraphidium arcuatum		■	■									
Monoraphidium contortum				■	■					■	■	■
Monoraphidium komarkovae									■			
Monoraphidium minutum						■				■		
Nephrocytum agardhianum				■								
Oocystis borgei	■	■	■	■	■	■	■	■	■	■	■	
Phacotus sp.			■	■	■		■	■		■		■
Planctonema lauterbornii			■						■	■	■	■
Quadrigula chodatii				■								
Scenedesmus acuminatus				■								
Staurastrum pingue												■
Staurastrum planctonicum										■		
Staurastrum sp.											■	
Staurastrum tetracerum										■		
Tetraëdron minimum		■	■	■	■				■	■	■	■
Tetrastrum komarekii				■								
Tetrastrum sp.												
Tetrastrum staurogeniaeforme		■		■		■	■	■				
Treubaria planctonica									■			
Willea vilhelmii						■						
**Prymnesiophyceae**												
Phaeocystis sp.				■								
Leucocryptos sp.	■	■	■							■	■	

where: the presence of individual taxa in the sample was marked in green.

Table S2. List of species detected in ŁL in controls and the experiments after 7 days of exposition to the exudates from *Synechococcus* sp. in study period.

**Present Taxa**	**Control**				**Experiment**			
	**May**	**June**	**July**	**August**	**May**	June	July	August
**Cyanophyceae**								

Aphanocapsa holsatica	+	+	+	+	+	+	+	+
Aphanocapsa incerta	+	+	+	+	+	+	+	+
Aphanothece spp.					+			
Chroococcus limneticus		+						
Cyanodictyon imperfectum						+		
Cyanodictyon planctonicum		+						
Limnothrix redekei								+
Snowella atomus	+				+		+	
Snowella lacustris		+						
Synechococcus sp.							+	
Woronichinia naegeliana				+	+			+
**Chrysophyceae**								
Dinobryon divergens	+				+			
**Bacillariophyceae**								
Cyclotella cyclopuncta			+					
Cyclotella radiosa		+	+	+		+		+
Fragilaria construens.		+						
Gomphonema sp.				+				
Navicula sp.								+
Nitzschia paleacea			+	+				
Nitzschia spp.			+					
Odontella mobiliensis								+
**Charophyceae**								
Cosmarium subcostatum							+	
**Euglenophyceae**								
Trachelomonas volvocina			+				+	
**Chlorophyceae**								
Coelastrum microporum			+	+				
Desmodesmus communis			+			+		
Desmodesmus intermedius							+	
Monoraphidium contortum	+			+				
Monoraphidium minutum		+	+			+	+	+
Oocystis borgei		+	+		+		+	
Scenedesmus acuminatus							+	
Tetraëdron minimum				+			+	
Tetrastrum staurogeniaeforme	+	+	+	+	+	+	+	

where: the presence of individual taxa in the sample was marked in green.

Table S3. List of species detected in RL in controls and the experiments after 7 days of exposition to the exudates from *Synechococcus* sp. in study period.

Present taxa	Control				Experiment			
	May	June	July	August	May	June	July	August
**Cyanophyceae**								
Aphanocapsa holsatica	+	+	+	+	+	+	+	+
Aphanocapsa incerta	+	+	+	+	+	+	+	+
Lemmermanniella pallida	+							
Microcystis spp.					+			

Planktolyngbya limnetica		■	■			■	■	
Pseudanabaena spp.		■				■		
Snowella atomus		■						
Snowella lacustris		■					■	
**Synurophyceae**								
Mallomonas caudata	■				■			
**Bacillariophyceae**								
Amphora pediculus		■				■		
Aulacoseira granulata				■				■
Cyclotella comensis	■							
Cyclotella cyclopuncta		■	■	■				
Cyclotella radiosa								■
Navicula sp.		■	■			■		■
Pinnularia sp.		■				■		
Ulnaria delicatissima		■						
**Charophyceae**								
Koliella longiseta f. *longiseta*		■				■		
**Euglenophyceae**								
Trachelomonas hispida	■				■			
**Chlorophyceae**								
Ankistrodesmus falcatus						■		
Coelastrum microporum		■				■		■
Crucigenia tetrapedia		■	■				■	■
Crucigeniella apiculata		■						
Desmodesmus communis		■	■			■		■
Dictyosphaerium pulchellum						■		
Monoraphidium contortum		■				■		
Monoraphidium komarkovae		■				■	■	
Monoraphidium minutum		■	■			■	■	
Oocystis borgei		■	■	■		■	■	■
Phacotus sp.	■	■	■	■	■	■	■	■
Planctonema lauterbornii	■	■	■	■	■	■	■	■
Sphaerocystis schroeteri		■						
Tetraëdron caudatum	■	■						
Tetraëdron minimum		■						
Tetrastrum globrum						■		
Tetrastrum komareki		■		■			■	■
Tetrastrum spp.					■			
Tetrastrum staurogeniaeforme		■	■			■	■	

where: the presence of individual taxa in the sample was marked in green.

Table S4. List of species detected in ŻL in controls and the experiments after 7 days of exposition to the exudates from *Synechococcus* sp. in study period.

Present taxa	Control				Experiment			
	May	June	July	August	May	June	July	August
**Cyanophyceae**								
Aphanocapsa delicatissima				■	■			

Aphanocapsa holsatica	■	■	■	■	■		■	■
Aphanocapsa incerta	■			■	■			
Aphanothece parallelliformis					■			
Cyanodictyon imperfectum			■					
Planktolyngbya limnetica								■
Pseudanabaena limnetica					■			
Snowella atomus		■		■		■		
Woronichinia naegeliana					■			
**Cryptophyceae**								
Plagioselmis spp.					■			
**Bacillariophyceae**								
Achnanthes spp.				■				
Amphora libyca		■				■		
Amphora pediculus				■				
Cyclotella radiosa	■	■	■	■	■	■	■	■
Fragilaria construens.		■						■
Fragilaria pinnata						■		
Navicula pygmaea		■						
Navicula sp.		■					■	
Nitzschia paleacea		■		■				
Stauroneis phoenicenteron								■
Ulnaria delicatissima				■	■			■
**Charophyceae**								
Koliella longiseta f. *longiseta*	■	■	■	■	■		■	■
**Chlorophyceae**								
Coelastrum astroideum	■	■						
Coelastrum microporum		■				■		■
Coenocystis planctonica		■	■	■	■	■		
Crucigenia tetrapedia		■	■	■			■	
Desmodesmus communis	■	■	■		■	■	■	■
Desmodesmus intermedius					■			
Monoraphidium contortum	■	■	■		■	■	■	■
Monoraphidium komarkovae						■		
Monoraphidium minutum	■	■	■	■	■	■	■	■
Oocystis borgei				■	■	■	■	
Pediastrum tetras					■			
Scenedesmus acuminatus		■				■	■	
Tetraëdron caudatum			■					
Tetraëdron minimum	■	■	■	■		■	■	■
Tetrastrum spp.	■	■						
Tetrastrum staurogeniaeforme		■	■	■		■	■	

where: the presence of individual taxa in the sample was marked in green.
